# Prevalence, Intensity, and Risk Factors for Helminth Infections in Pigs in Menoua, Western Highlands of Cameroon, with Some Data on Protozoa

**DOI:** 10.1155/2022/9151294

**Published:** 2022-05-10

**Authors:** Marc K. Kouam, Fabrice D. Ngueguim

**Affiliations:** ^1^Department of Animal Sciences, Faculty of Agronomy and Agricultural Sciences, PO BOX 188, Dschang, Cameroon; ^2^Center for Research on Filariases and Other Tropical Diseases (CRFilMT), P.O. Box 5797, Yaoundé, Cameroon

## Abstract

Helminthes affect satisfactory pig farming by causing poor growth rate and infertility. The objective of this study was to investigate the occurrence of helminthes in pig production, as well as factors influencing their prevalence in Menoua, Western Highlands of Cameroon. Thus, 597 fecal samples from 100 farms of three production types (farrower, grower, and farrow-to-finish) were collected together with data on farmer and management characteristics. Samples were qualitatively and quantitatively analyzed. Eggs of eight helminthes were identified: *Hyostrongylus rubidus* (81.10%, 50-550 epg), *Strongyloides ransomi* (34.5%, 50-150 epg), *Trichostrongylus* sp. (28.1%, 50-650 epg), *Ascaris suum* (11.6%, 50-200 epg), *Metastrongylus* sp. (10.4%, 50-250 epg), *Oesophagostomum dentatum* (5.7%, 50-150 epg), *Trichuris suis* (4.0%, 50-150 epg), and *Macracanthorhynchus hirudinaceus* (0.2%, 50-50 epg). The overall prevalence was 89.3% (533 out of 597). Single infestations were 30.2%, while mixed infestations were 59.1%. *A. suum*, *S*. *ransomi*, and strongyles (*H. rubidus*, *Trichostrongylus* sp., *Metastrongylus* sp., and *O. dentatum*) were found in almost all age groups but the prevalence of *A. suum* increased with the growing age to drop in older animals. *H. rubidus* was found in all farm types followed by *S. ransomi* in farrower and farrow-to-finish farms. The other parasites were present only in farrow-to-finish farms. Coccidia parasites were also found including *Isospora suis* (26.30%, 50-12500 oocysts per gram of feces (opg)) and *Eimeria* spp. (1.40%, 100-100 opg). The risk of infestation for some parasites was lower with increasing herd size, high education level of farmers, and in wooden piggeries and semipermanent structures. The infestation risk was higher for all the investigated parasites for pigs escaping the pens. The overall significance of these parasites on growth and reproduction of the naturally infested pigs deserve assessment. Necropsy studies to confirm the worm burden are needed. Risk factors were identified, thus paving the way to design successful helminth control in pig production enterprises.

## 1. Introduction

Food insecurity is among the greatest challenges of the twenty-first century, especially in developing countries where poverty is rampant. In these countries, the pressing and increasing demand for animal protein is exacerbated by the high population growth and the soaring urbanization [1]. In Cameroon, the per capita meat consumption is estimated as 12.7 kilogram/inhabitant/year (kg/inh/year) far below 33 kg/inh/year as recommended by the Food and Agricultural Organization of the United Nations (FAO) [2]. Of the 12.7 kg/inh/year, pork only contributes 2.02 kg/inh/year [3], which is very low. Diseases including parasitic infestations are among the limiting factors of pig production in the country [3].

Among parasites, gastrointestinal parasites (GIP) hinder a profitable pig production in many ways. They injured some vital organs which play a key role in metabolic activities [4] and affect the wellbeing of pigs by robbing the essential nutrients required for optimum reproduction and productivity [5]. In pig production, the manifestation of pig parasitism includes anorexia, poor growth rate, anemia, emaciation, infertility, and condemnation of affected organs after slaughter [4, 6–8]. Severe cases of helminthiasis in young pigs have been associated with diarrhea, loss of electrolytes, and death [9]. Lung parasites also impair pig productivity as noticed with the lungworm *Metastrongylus* spp. which cause coughing, poor growth, emaciation, inappetence, and enhancement of other diseases [10, 11].

Knowledge on pig parasitism is of paramount importance in order to design appropriate control strategies to suppress the deterring impact of parasitism on productivity. However, in Cameroon, data on pig parasitism is very limited, with one report in 2018 [12] and another one dating back to 17 years ago, presenting the prevalence of GIP of pigs in the country [13]. Thus, the objectives of this cross-sectional study were to determine the prevalence and intensity of helminthes occurring in the Western Highlands of Cameroon and to evaluate the risk factors associated with these parasite infestations.

## 2. Material and Methods

### 2.1. Study Area

**Figure 1:**
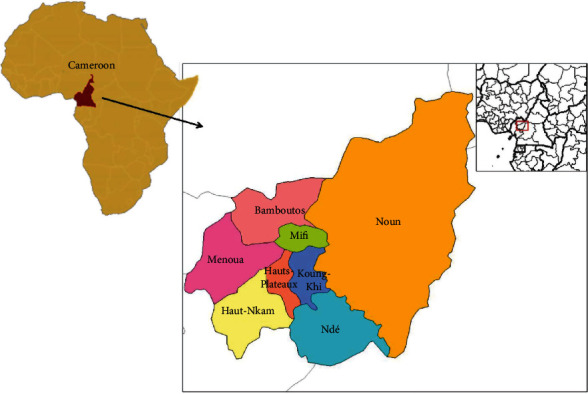
Map of the West region of Cameroon showing Menoua Division.

The study was carried out from May to September 2016 in Menoua Division in West region of Cameroon (Figure 1). The area lies between longitude 9°49′-10°20′ East of the Greenwich meridian and latitude 5°17′-6°22′ North of the equator. The region is characterized by a typical climate with two main seasons, the dry season ranging from November to mid-March and the rainy season which prevails from mid-March to October. Temperature ranges between 15° and 24°C [14]. Animal husbandry in the division consists of rearing small and large ruminants, cavies, pigs, rabbits, broilers, and layers, among others. The West region is one of the highest pig production regions of the country [3].

### 2.2. Study Design and Sample Size Determination

The study was a cross-sectional investigation. Pig farms were randomly selected, and the snowball sampling technique was used to identify pig farms due to the absence of farmer's registers in the veterinary health authorities' office of the West region. The snowball technique consists of using information from the first identified farmer to locate the next farmer. All pigs were sampled in the piggery if the population size was less than 8 animals. Above eight animals on a farm, a maximum of ten animals were randomly sampled. Pregnant sows and piglets below 2 months old were excluded from the study, as well as farms which had received an anthelmintic treatment within 2 months before the study (prepatent period of most parasites less than two months). Animals were categorized as piglets (2–6 months), growers (6-12 months), young adults (12–18 months), middle-aged adults (18-24 months), and older adults (more than 24 months). The farm types on the field were farrower, grower, and farrow-to-finish farms. Farrowing farms were those specialized in piglet production; grower farms were those specialized in fattening pigs for meat purposes, while farrow-to-finish farms produce piglets for sale and also fatten pigs for meat.

The sample size was computed based on the formula for sample size calculation [15] as follows: *n* = *Z*^2^*P* (1–*P*)/*d*^2^, where *n* is the required sample size, *Z* is the normal deviate (1·96) at the 5% level of significance, *P* is the estimated prevalence of infection in pigs (50%) in the absence of the previous data, and *d* is the allowable error of estimation or precision (0·05). Thus, the computed sample size (*n*) was determined as 385. However, the total number of sampled animals was increased depending on the economical means to 597 to increase the power of the test statistics.

### 2.3. Fecal Samples and Data Collection

Farms were visited early in the morning. Each farm owner was asked to gently hit the pigs' back to make them defecate. Immediately after defecation, the topper layer of the stool that had not touched the ground was collected with gloved hands and introduced into a screw cap container containing 10% formalin. Data on herd characteristics, herd management practices, and farmer status were collected through a questionnaire. The investigators completed the questionnaires by interviewing the farmers at the time of fecal samples collection.

### 2.4. Fecal Sample Analysis

Fecal samples were analyzed qualitatively and quantitatively using the saturated salt solution (NaCl) as flotation fluid (specific gravity = 1.2). The simple flotation method was used to detect the parasite eggs and oocysts which were identified microscopically based on morphology and size [11, 16, 17]. The Modified McMaster [17] test, with a sensitivity of 50 eggs per gram of feces (epg), was used to estimate the parasitic burden in the individual pig fecal samples.

Heavy eggs (from trematode, *Metastrongylus*, and Acantocephalan parasites) were screened using the simple sedimentation test, as described by Zajac and Conroy [17]. Slides were mounted and examined at 100 and 400 magnifications.

An animal was considered infected with a parasite if at least one egg/oocyst was detected in the flotation solution.

### 2.5. Statistical Analysis

The prevalence was presented in terms of percentage, whereas the epg was presented in terms of mean and standard deviation. The differences in the mean egg counts (epg) between parasites were tested using the Mann-Whitney *U* tests.

The relationship between the prevalence of various infections and the investigated factors was analyzed by defining a binary outcome variable where positive samples were coded as 1, while negative samples were coded as 0. The risk factor analysis was performed only for parasites with prevalence greater than 10%.

The univariate logistic regression was used to analyze the association of all examined factors (animal, farm, and farmer-related factors) as independent variables with the prevalence of each infestation. Variables significant at *p* < 0.05 were tested for multicollinearity and selected for inclusion in the multivariate logistic regression model. Multicollinearity was assessed by evaluating the standard errors of the logit coefficients, a standard error > 2 being indicative of a numerical problem. Thus, factors with a standard error > 2 were dropped, and the model was run for a second time. Overall fit of the logistic regression models was assessed using the Hosmer–Lemeshow goodness-of-fit statistics. Results were presented as adjusted odds ratios (OR) with 95% confidence intervals (CI). A *p* value of <0·05 was considered significant. All statistics were performed using the SPSS statistical package (version 13.0, SPSS Inc., USA).

## 3. Results

Eggs of eight helminthes were identified out of the 597 fecal samples examined (Table 1). The overall prevalence of helminth infestations was 89.3% (533 out of 597 animals). Single infestations were 30.2%, while mixed infestations were 59.1% composed of dual (35.5%), triple (16.9%), quadruple (5.5%), and quintuple (1.1%) infestations. Pigs predominantly shed eggs of *Hyostrongylus rubidus* (81.10%), followed by those of *Strongyloides ransomi* (34.5%), *Trichostrongylus* sp. (28.1%), and *Ascaris suum* (11.6%). The eggs shed to a lesser extend were those of *Metastrongylus* sp. (10.4%), *Oesophagostomum dentatum* (5.7%), *Trichuris suis* (4.0%), and *Macracanthorhynchus hirudinaceus* (0.2%). Among the most predominant helminthes found, the mean epg for *H. rubidus* (88.8 ± 70.4) was significantly higher than the mean epg for *S. ransomi* (54.4 ± 16.5), *Trichostrongylus* sp. (70.7 ± 64.7), and *A. suum* (75.4 ± 48.2), whereas the mean epg for *A. suum* was significantly greater than the mean epg for *S. ransomi* (Table 1).

*H. rubidus* and *S. ransomi* were found in all age group but tended to be predominant in older adults (89.5% and 68.4%, respectively), while the prevalence of *A. suum* increased with the growing age but dropped in older pigs (from 24 months upwards) down to 10.5% (Table 2). *Trichostrongylus* sp. and *Metastrongylus* sp. were found in almost all age group without a particular trend. *O. dentatum* was mostly found in piglets and older pigs, while *T. suis* was predominant in growing pigs (Table 2). *H. rubidus* was found in all farm types (growers, farrowers, and farrow-to-finish) followed by *S. ransomi* in farrower and farrow-to-finish farms. The other parasites were present only in farrow-to-finish farms.

Several risk factors (herd size, contact with other domestic animals, education level of farmer, housing, pig age, and farmer gender and age) were found to be significantly associated (*p* < 0.05) with *H. rubidus*, *S. ransomi*, *Trichostrongylus* sp., and *A. suum* infestations in pigs. The risk of *H. rubidus* infestations in pigs diminished by 0.95 for a unit increase in herd size and was 5.03 times higher in pigs in contact with other domestic animals than in those not in contact with other domestic animals (Table 3). The risk for *S. ransomi* was 0.13 and 0.19 times lower for pigs owned by farmers who had attained higher and secondary school compared with farmers who never went to school; this risk was also 0.15 and 0.18 times lower for pigs raised in wooden piggeries and semipermanent structures, respectively, than for pigs in permanent structures. But this risk was nearly 2.5-fold greater in pigs in contact with other domestic animals than in pigs not in contact with other domestic animals (Table 4). The risk of *Trichostrongylus* sp. increased by 1.04 as the pig age increased by 1 month and was 2.45-fold higher in pigs owned by men compared with women. This was 0.39 lower but 5-fold greater in wooden piggeries and semipermanent structures compared with permanent structures (Table 5). There was a 1.06 increase in the risk for *A. suum* infestations in pigs as the farmer's age increased by 1 year. There was a 4-fold increase in the risk of *A. suum* infestation if pigs were in contact with other domestic animals in comparison with if they were not (Table 6).

Coccidia parasites were also found including *Isospora suis* (26.30%, 106.03 ± 71.38 oocysts per gram of feces (opg)) and *Eimeria* spp. (1.40%, 100 ± 00 opg).

## 4. Discussion

The animal sampled were shedding eggs of eight helminthes. In general, eggs of *Hyostrongylus*, *Oesophagostomum*, and *Globocephalus* are reported to be indistinguishable. However, *H. rubidus* and *O. dentatum* eggs are readily identifiable, as previously described [11, 16]. Indeed, *Globocephalus urosubulatus* eggs are approximately 50 − 56 × 26 − 35 *μ*m, while the size of *H. rubidus* and *O. dentatum* are 69 − 85 × 39 − 45 *μ*m and 66-80 × 38-47 *μ*m, respectively. *O. dentatum* eggs contain 8 to 16 cells, while those of *H. rubidus* contain at least 32 cells [16]. *G. urosubulatus* egg contains 6 to 8 cells [16]. The species identified are in line with the results of a past work in the same area [13] where six parasite families were identified, suggesting that these parasites have become endemic and that no control strategy is available or has been successful.

The helminthes found in this study to infest pigs are all of economic importance, even though the pathogenic effect of some are more pronounced and cause more serious deleterious effect in pig production. Strongylid parasites of pigs such as *Hyostrongylus*, *Oesophagostomum*, and *Trichostrongylus* found in the present work are known to cause light clinical manifestations, except in heavy infestations [18]. Heavy infestations with *H. rubidus* cause ulcerative gastritis resulting in anemia and reduced production while that with *Oesophagostomum* cause nodule development in the intestinal wall leading to enteritis and reduced production [17]. Infestations with *Trichostrongylus* have been reported to cause gastroenteritis in their host leading to weight loss, diarrhea, and poor growth rate [11]. Heavy infestations with *S. ransomi* have been documented as the cause of bloody diarrhea, anemia, clinical respiratory diseases, vomiting, and sudden death [11, 17]. Low levels of infestation with *A. suum* have been found to cause inappetence and poor feed conversion [9] resulting in reduced growth; they also cause liver condemnation and predispose pigs to bacterial or viral pneumonia [17]. Infestations with *T. suis* can cause diarrhea, anorexia, anemia, poor growth, dehydration, dysentery, emaciation, and death (Beer and [9, 19, 20]). Heavy infestation with *Metastrongylus* in young pigs cause coughing, dyspnea, and nasal discharge but in case of secondary bacterial infection, inappetence and weight loss may also occur [11]. *M. hirudinaceus* causes damage to the intestinal wall, inducing diarrhea and weight loss [11]. A single case of the latter parasite was recorded, suggesting that its distribution was limited and that it was not a threat to pig production in the area. Nevertheless, the very low prevalence recorded in this study might be underestimated since the coprological sedimentation test was reported to be a poor predictor of both prevalence of infestation and the real parasite burden due to the high number of false negative results (prevalence reduced from 61 to 16% in wild boar) [21].

The overall prevalence was high, as well as mixed infestations, suggesting a poor productivity in pig husbandry in the study area as the result of the deleterious effect of these multiples parasites in pigs. Though the infestation level was low (<500epg) to moderate (<2000epg) for each individual parasite [11, 22], the combined effect of these parasites probably lead to reduced productivity.

The protozoan found in the study area are of importance in pig production. Both *Isospora* and *Eimeria* are known to cause diarrhea, weight loss, unthriftiness, dehydration, and death in piglets but to be less pathogenic in adult pigs [10, 11], suggesting that in the study area, pregnant sows should be treated before and after farrowing; furthermore, after the first treatment, farmers should transfer the pregnant sows about to give birth from the pregnancy box to a clean maternity box to keep the piglets from infection.

The negative effect of multiparasitism on animal health is manifold due to interactions (either synergistic or antagonistic) among parasites and between parasites and microbial agents: coinfections have an influence on the clinical signs, duration, and treatment of diseases (Vaumourin et al., [23]). For instance, (1) coinfection, in addition to altering the likelihood of parasite establishment, growth, and shedding of involved parasites, can generate super-shedders (i.e., individuals that for a period of time yield many more infective stages than most other infected individuals of the same host species) [24]; (2) during coinfection, one parasite can be a driver of outbreaks of other disease agents [25] as has been demonstrated in zebra where gastrointestinal helminthes were shown to alter the dynamics of anthrax (*Bacillus anthracis*) by rendering hosts less able of mounting effective anti-anthrax immune responses during the rainy season [26]. Alternatively, coinfection can have positive effects on the host, ranging from the inhibition of the growth of certain parasites to the reduction of host mortality. For example, a recent study on the effect of coinfection with intestinal parasites on COVID-19 severity showed that patients coinfected with parasites had lower odds of developing severe COVID-19, and all the deaths were among patients without parasites [27]. Though such investigations have not yet been done in pigs, it is obvious that multiparasitism increase the severity of individual parasites because of their combined effect on the immune system. However, because of the occurrence of deathly diseases in the study area such as African swine fever, erysipelas, and colibacilosis (Kouam, unpublished data), future research should focus on both the negative and positive effect on the pig host, of co-infection with intestinal parasites and with other microbial agents.

The high overall prevalence observed here is in disagreement with past studies in Africa where the highest prevalence of helminthes was 58 and 70.5% in free range pigs, respectively, in Zimbabwe [6] and Nigeria [28]. It also disagrees with a former study in Ghana on intensively managed pigs which recorded total helminthes prevalence not greater than 28% [29]. However, the overall prevalence is similar to a former record (97.6%) obtained in the same area two decades ago [13]. It is admitted that intensively managed pigs harbor less parasites than free range or extensively managed pigs [29] not often dewormed or reinfected. The pigs sampled in our study are normally penned, though some often escape. Thus, the high prevalence recorded in the study is probably related to poor biosecurity, management, and hygiene practices as previously documented (Kouam et al., unpublished data). *H. rubidus* recorded the highest prevalence in this study (81.90%). This is lower than those documented by Tamboura et al. [30] in Burkina Faso, Obonyo et al.[31] in Kenya, and Aiyedun and Oludairo [32] in Nigeria who reported a prevalence of 11, 22, and 55%, respectively. Aiyedun and Oludairo [32] also reported *H*. *rubidus* to record the highest prevalence and egg load in Nigeria's North Central State of Kwara. Nevertheless, our result disagree with the previous findings in sub-Saharan Africa as in Burkina Faso (Tamboura et al. [30] or Plateau state, Nigeria [28] where *A. suum* had the highest prevalence of 40 and 18.5%, respectively. The predominance of *H. rubidus* is related to the moist condition prevailing in piggeries of the study area, which is conducive to the completion of the short, direct life cycle of this parasite.

Parasitism is often associated with host age. *A. suum*, *S. ransomi*, and strongyles (*H*. *rubidus*, *Trichostrongylus* sp., *Metastrongylus* sp., and *O. dentatum*) were found in almost all age groups. This is in line with other findings [29, 33]. The lowest prevalence of *A. suum* was recorded in piglets, which disagrees with the report by Obonyo et al. [31] in which no parasite was documented in piglets; it is also in disagreement with the report by Kagira [34] in which piglets recorded the highest prevalence of infestation with *A. suum*, while growers recorded the least prevalence. The variation in the age of piglets and growers sampled in different studies is probably the reason for the discrepancy in the results obtained in these studies. Obonyo et al. [31] classed piglets as animals below 3 months and growers as those between 3 and 5 months, whereas piglets in this study were considered as animals below 5 months. While piglets below 3 months would hardly shed *A. suum* egg, those aged 5 months can allow this parasite to complete the life cycle and start laying egg. *T. suis* was the only species not recorded in older pigs (>18 months); this is probably linked to the highly immunogenic properties of this parasite influencing its distribution in young animals and pregnant sows rather than in older animals [35, 36].

Most of farms sampled were farrow-to-finish farms. Indeed, very few farms in the study were specialized in a particular production type probably due to the local economic context of pig industry. As reflected by sample size of different farm types, farrower and grower farms were very scant in the study area. Apart from *H. rubidus* that was recorded in all farm types, all the helminthes were found only in farrow-to-finish farms. The latter farm type is ideal for parasites to remain endemic since transmission is sustained when pregnant sows are not removed from the contaminated area before farrowing and during suckling; with the prevailing moist in piggeries, especially in tropical areas, the free living stages of most parasites will survive in the environment for a very long time, so as to be able to re-infest piglets; in addition most of the parasites recorded have direct life cycle with eggs that may survive after 3 to 4 years as in *T. suis* [11] or 5 years as in *A. suum* (Mornet et al.,[37]). Another reason is the increase in fecal egg count contaminating the environment which occurs when hypobiotic larvae resume their development during the periparturient relaxation of immunity. Thus, the high, total prevalence of helminthes observed in the study may be justified also by the importance of farrow-to-finish farms adopted by farmers.

Several risk factors were identified in this study. Herd size and contact with other domestic animals were associated with infestation with *H. rubidus*. The decrease in the risk of infestation observed with the increasing herd size may be explained by the improved hygienic, management, and biosecurity measures enforced by farmers to reduce the disease risk and secure the heavy investment. Indeed, keeping a large herd require important financial inputs which the farmer would like to secure through better care for animals. Given that the life cycle of this parasite is short, implementation of these measures would have positive and immediate result on *H. rubidus* infestations. The significantly higher risk of *H. rubidus* infestation in pigs coming into contact with other domestic animals is due to the fact that, as pigs escape the pen from time to time, they come into contact with the environment probably contaminated with larvae, since the L_3_ larvae after leaving the feces under favorable condition would migrate on to the herbage where they are easily ingested [11]. A substratum (straw, herbage) for the larvae to migrate is not always available in piggeries, except when animals are fed kitchen waste or forage. Our finding is similar to that of Kochanowski et al. [38] who found that the risk for parasites occurrence was higher in pig farms with paddock. Farmer education, housing, and contact with other domestic animals were the factors significantly influencing the prevalence of *S. ransomi*. Farmers with higher and secondary level education had pigs with lower *S. ransomi* infestation compare with farmer who never went to school. This is probably the results of these educated farmers having better skills in the daily management and adoption of hygienic measures required to interrupt this parasite life cycle. The risk of infestation was significantly lower in wooden piggeries and semipermanent structures compared with permanent structures. This finding might be explained by the life cycle of this parasite which is more likely to be completed in permanent structures rather than in wooden piggeries and semipermanent structures often destroyed and rebuilt. In the process of renewing the structure, animals are transferred from the contaminated area to a new uncontaminated environment of the newly built lodging. The higher risk of infestation in pigs in contact with other domestic animals is probably due the fact that, as pigs escape, they probably become infested with environmental infective larvae accumulated under the moisture which is common in the tropics. Our result agrees with a previous report in Poland [38]. Pig age, farmer gender, and housing significantly affect the prevalence of *Trichostrongylus* sp. infestations. The increasing risk of infestation with the growing pig age is probably due to a lower immunity of pigs which in farrow-to-finish farms were mostly females. Immunity to *Trichostrongylus* is slowly acquired but would wane throughout the periparturient period when hormonal changes during late pregnancy and lactation lower the host resistance to parasites, which subsequently result in the establishment of higher worm burdens [39]. Pigs managed by men had a two-fold risk of infestations compared with pigs managed by women because men usually did not only rear pigs but also kept other domestic animals. Thus cross-transmission was possible for *Trichostrongylus* sp. which is a multiple-host species, between pigs and other domestic animals kept by men such as sheep and goats. Farmer age and contact with domestic animals significantly affect prevalence of *A. suum*. As the farmer age increased, the risk of infestation with this parasite was also increased. *A. suum* is thought to be zoonotic [11, 40, 41] and likely to be transmitted from pig to man and vice versa. Thus, since pig keeping was mostly adults' activity rather than youth's, close contact between pigs and adults might have led to zoonotic transmission of *A. suum*, such that keepers (adults) have become the reservoir of this parasite. It is worth mentioning that people, though having toilet facilities at home, often defecate in the bush when they happen to be out and far from their house. Pigs therefore might ingest *A. suum* eggs of human origin from contaminated forage harvested in the bush. Pig age has been shown to have an effect on *A. suum* prevalence in Danish pig herds [42]; this is in contrast to our result and may be explained by variations of the age of pigs sampled. Kochanowski et al. [38] investigated the association between herd size and the prevalence of *A. suum*. Their results disagree with ours and may be explained by the differences in management and production system. Contact with other domestic animals appear as a risk factor because escaped pigs are more likely to become contaminated outside the piggery since *A. suum* egg is very resistant in the environment [17].

In conclusion, this study demonstrated the extent of helminthes occurrence in pig production in Menoua in the Western Highlands of Cameroon. The helminthes spectrum was made up of at least eight species occurring at various prevalence and intensity. Their total prevalence was high, and their intensity moderate indicating subclinical infections. In spite of the moderate intensity of each individual parasite, the combined effect of all these parasites probably reduces productivity. Future necropsy studies to confirm the worm burden in pigs will enlighten our understanding of the statute of pig parasitism in the West region. The overall significance of these parasites on growth and reproduction of the naturally infested pigs deserve assessment. Risk factors influencing parasite infestations were identified, thus paving the way to the design of successful helminth control in pig production enterprises.

## Figures and Tables

**Table 1:** Prevalence (%) and intensity of helminthes in pigs (*N* = 597) in Menoua.

Helminthes	Prevalence			Intensity	
	n	%	95% CI	Mean epg ± Sd	Range
*Hyostrongylus rubidus*	489	81.90	78.5-84.9	88.8 ± 70.4^a,b,c^	50-550
*Strongyloides ransomi*	206	34.5	30.7-38.5	54.4 ± 16.5^a,d,e^	50-150
*Trichostrongylus* sp.	168	28.1	24.6-32	70.7 ± 64.7^b,d^	50-650
*Ascaris suum*	69	11.6	9.2-14.5	75.4 ± 48.2^c,e^	50-200
*Metastrongylus* sp.	62	10.4	8.1-13.2	60.5 ± 31.5	50-250
*Oesophagostomum dentatum*	34	5.7	4-7.9	61.7 ± 32.7	50-150
*Trichuris suis*	24	4.0	2.6-6.0	54.2 ± 20.4	50-150
*Macracanthorhynchus hirudinaceus*	1	0.2	0.0-1.1	50 ± 0	50-50

n = number of infested pigs; CI = confidence interval; Sd = standard deviation; epg = egg per gram of feces; ^a,b,c,d,e^Mean epg with same superscripts are significantly different.

**Table 2:** Helminth infestation according to age class (month) of pigs and farm type in Menoua.

	*H. rubidusn*(%)	*S. Ransomin*(%)	*T.* sp. *n* (%)	*A. suumn*(%)	*M.* sp. *n* (%)	*O. dentatumn*(%)	*T. suisn*(%)
*Age class*							
[1-6[(*N* = 303)	262 (86.5)	115 (38.0)	87 (28.7)	27 (8.9)	32 (10.6)	23 (7.6)	10 (3.3)
[6-12[(*N* = 177)	132 (74.6)	40 (22.6)	34 (19.2)	119 (10.7)	9 (5.1)	4 (2.3)	6 (3.4)
[12-18[(*N* = 80)	64 (80.0)	34 (42.5)	25 (31.3)	17 (21.3)	10 (12.5)	3 (3.8)	20 (10.0)
[18-24[(*N* = 18)	14 (77.8)	4 (22.2)	16 (88.9)	4 (22.2)	7 (38.9)	0 (0.0)	0 (0.0)
[24-36[(*N* = 19)	17 (89.5)	13 (68.4)	6 (31.6)	2 (10.5)	4 (21.1)	4 (21.1)	0 (0.0)
*Farm type*							
Grower (*N* = 4)	4 (100)	0 (0.0)	0 (0.0)	0 (0.0)	0 (0.0)	0 (0.0)	4 (0.0)
Farrower (*N* = 8)	7 (87.5)	7 (87.5)	0 (0.0)	0 (0.0)	0 (0.0)	0 (0.0)	1 (12.5)
Farrow-to-finish (*N* = 584)	417 (81.7)	199 (34.1)	167 (28.6)	69 (11.8)	62 (10.6)	34 (5.8)	19 (3.3)

*N* = number of samples examined; *n* = number positive; (%) = prevalence in percentage; *H. rubidus = Hyostrongylus rubidus*; *S. ransomi = Strongyloides ransomi*; *T.* sp. *= Trichostrongylus* sp.; *A. suum = Ascaris suum*; *M.* sp*. = Metastrongylus* sp.; *O. dentatum = Oesophagostomum dentatum*; *T. suis = Trichuris suis*.

**Table 3:** Logistic regression analysis of risk factors for *Hyostrongylus rubidus* infection in pig farms in Menoua Division. Results are expressed in terms of odd ratio (OR) and 95% confidence interval (CI).

Factor				Univariate analysis		Multivariate analysis		
	*N*	*n*	(%)	OR	*p* value	OR	95% CI	*p* value
*Continuous variables*								
Farmer age (year)	597	489	81.9	0.98	0.17			
Pig age (month)	597	489	81.9	0.97	0.12			
Herd size	597	489	81.9	0.94	0.01	0.95	0.91-0.98	0.01∗
*Categorical variables*								
*Farmer gender*								
Male	488	403	82.6	1.26	0.36			
Female	109	86	78.9	1.00				
*Farmer education level*								
Higher education	51	45	88.2	0.00	0.99			
Secondary school	378	298	78.8	0.00	0.99			
Primary school	158	136	86.1	0.00	0.99			
Never schooled	10	10	100.0	1.00				
*Animal breed*								
Crossed breed	547	444	81.2	0.00	0.99			
Duroc	10	10	100.0	1.00	1.00			
Landrace	2	0	0.0	0.00	0.99			
Large white	35	33	94.3	0.00	0.99			
Pietrain	2	2	100.0	1.00				
Naima	1	0	0.0	—				
*Housing*								
Wooden piggery	525	426	81.1	0.00	0.99			
Semipermanent structure	39	30	76.9	0.00	0.99			
Permanent structure	33	33	100.0	1.00				
*Floor type*								
Cemented	201	162	80.6	0.87	0.55			
Non cemented	396	327	82.6	1.00				
*Contact with domestic animals*								
Yes	208	202	97.1	11.96	0.01	5.03	2.07-12.18	0.01∗
No	389	287	73.8	1.00		1.00		
*Contact with wild life*								
Yes	545	441	80.9	0.35	0.05			
No	52	48	92.3	1.00				

*N* = number of samples examined; *n* = number positive; (%) = prevalence in percentage. ∗ = significant *p* value.

**Table 4:** Logistic regression analysis of risk factors for *Strongyloides ransomi* infection in pig farms in Menoua Division. Results are expressed in terms of odd ratio (OR) and 95% confidence interval (CI).

Factor				Univariate analysis		Multivariate analysis		
	*N*	*n*	(%)	OR	*p* value	OR	95% CI	*p* value
*Continuous variables*								
Farmer age (year)	597	206	34.5	1.00	0.26			
Pig age (month)	597	206	34.5	1.01	0.36			
Herd size	597	206	34.5	0.92	0.01	0.97	0.93-1.02	0.36
*Categorical variables*								
*Farmer gender*								
Male	488	161	33.0	0.70	0.10			
Female	109	45	41.3	1.00				
*Farmer education level*								
Higher education	51	11	21.6	0.06	0.01	0.13	0.04-0.39	0.01∗
Secondary school	378	100	26.5	0.09	0.01	0.19	0.09-0.43	0.01∗
Primary school	158	87	55.1	0.30	0.14	—		
Never schooled	10	8	80.0	1.00		1.00		
*Animal breed*								
Crossed breed	547	181	33.1	0.52	0.06			
Duroc	10	8	80.0	4.23	0.09			
Landrace	2	0	0.0	0.00	0.99			
Large white	35	17	48.6	1.00				
Pietrain	2	0	0.0					
Naima	1	0	0.0					
*Housing*								
Wooden piggery	525	183	34.9	0.39	0.01	0.15	0.06-0.39	0.01∗
Semipermanent structure	39	4	10.3	0.08	0.01	0.18	0.03-0.90	0.03∗
Permanent structure	33	19	57.6	1.00		1.00		
*Floor type*								
Cemented	201	39	19.4	0.33	0.01	0.61	0.35-1.06	0.08
Non cemented	396	167	42.2	1.00		1.00		
*Contact with domestic animals*								
Yes	208	89	42.8	1.73	0.01	2.47	1.38-4.43	0.02∗
No	389	117	30.1	1.00		1.00		
*Contact with wild life†*								
Yes	545	175	32.1	0.32	0.01			
No	52	31	59.6	1.00				

*N* = number of samples examined; *n* = number positive; (%) = prevalence in percentage; † variable dropped in multivariate analysis due to collinearity. ∗ = significant *p* value.

**Table 5:** Logistic regression analysis of risk factors for *Trichostrongylus* sp. infection in pig farms in Menoua Division. Results are expressed in terms of odd ratio (OR) and 95% confidence interval (CI).

Factor				Univariate analysis		Multivariate analysis		
	*N*	*n*	(%)	OR	*p* value	OR	95% CI	*p* value
*Continuous variables*								
Farmer age (year)	597	168	28.1	1.00	0.66			
Pig age (month)	597	168	28.1	1.04	0.01	1.04	1.01-1.08	0.01∗
Herd size	597	168	28.1	1.06	0.01	1.02	0.98-1.06	0.22
*Categorical variables*								
*Farmer gender*								
Male	488	154	31.6	3.12	0.01	2.45	1.33-4.53	0.01∗
Female	109	14	12.8	1.00		1.00		
*Farmer education level*								
Higher education	51	0	0.0	0.00	0.99			
Secondary school	378	147	38.9	2.54	0.24			
Primary school	158	19	12.0	0.54	0.46			
Never schooled	10	2	20.0	1.00				
**Animal breed**								
Crossed breed	547	154	28.2	1.32	0.49			
Duroc	10	6	60.0	5.06	0.03			
Landrace	2	0	0.0	0.00	0.99			
Large white	35	8	22.9	1.00				
Pietrain	2	0	0.0					
Naima	1	0	0.0					
*Housing*								
Wooden piggery	525	122	23.2	0.41	0.01	0.39	0.18-0.83	0.01∗
Semipermanent structure	39	32	82.1	6.02	0.01	5.46	1.81-16.45	0.01∗
Permanent structure	33	14	42.4	1.00		1.00		
*Floor type*								
Cemented	201	73	36.3	1.80	0.01	0.85	0.54-1.35	0.49
Non cemented	396	95	24.0	1.00		1.00		
*Contact with domestic animals*								
Yes	208	63	30.3	1.17	0.39			
No	389	105	27.0	1.00				
*Contact with wild life*								
Yes	545	153	28.1	0.96	0.90			
No	52	15	28.8	1.00				

*N* = number of samples examined; *n* = number positive; (%) = prevalence in percentage; ∗ = significant *p* value.

**Table 6:** Logistic regression analysis of risk factors for *Ascaris suum* infection in pig farms in Menoua Division. Results are expressed in terms of odd ratio (OR) and 95% confidence interval (CI).

Factor				Univariate analysis		Multivariate analysis		
	*N*	*n*	(%)	OR	*p* value	OR	95% CI	*p* value
*Continuous variables*								
Farmer age (year)	597	69	11.6	1.02	0.03	1.06	1.02-1.09	0.01∗
Pig age (month)	597	69	11.6	1.04	0.03	0.99	0.93-1.06	0.95
Herd size	597	69	11.6	1.07	0.01	1.04	0.99-1.09	0.05
*Categorical variables*								
*Farmer gender*								
Male	488	42	8.6	0.28	0.01	1.90	0.23-15.12	0.54
Female	109	27	24.8	1.00		1.00		
*Farmer education level*								
Higher education	51	3	5.9	0.34	0.09			
Secondary school	378	42	11.1	0.69	0.19			
Primary school	158	24	15.2	1.00				
Never schooled	10	0	0.0					
*Animal breed*								
Crossed breed	547	60	11.0	0.49	0.11			
Duroc	10	2	20.0	1.00	1.00			
Landrace	2	0	0.0	0.00	0.99			
Large white	35	7	20.0	1.00				
Pietrain	2	0	0.0					
Naima	1	0	0.0					
*Housing*								
Wooden piggery	525	62	11.8	0.61	0.26			
Semipermanent structure	39	7	17.9	1.00				
Permanent structure	33	0	0.0					
*Floor type*								
Cemented	201	21	10.4	0.84	0.54			
Non cemented	396	48	12.1	1.00				
*Contact with domestic animals*								
Yes	208	36	17.3	2.25	0.01	3.98	1.64-9.62	0.01∗
No	389	33	8.5	1.00		1.00		
*Contact with wild life*								
Yes	545	65	11.9	1.62	0.36			
No	52	4	7.7	1.00				

*N* = number of samples examined; *n* = number positive; (%) = prevalence in percentage. ∗ = Significant *p*-value.

## Data Availability

All the data used for this work are included within this manuscript.
